# Vorasidenib for IDH-mutant grade 2 gliomas: clinical advances and future directions

**DOI:** 10.3389/fonc.2025.1628195

**Published:** 2025-06-27

**Authors:** Zhenjiang Pan, Jing Bao, Shepeng Wei

**Affiliations:** Department of Neurosurgery, Shidong Hospital, Shanghai, China

**Keywords:** Vorasidenib, astrocytoma, oligodendroglioma, IDH mutation, targeted therapy

## Abstract

Vorasidenib, a brain-penetrant dual inhibitor of mutant isocitrate dehydrogenase 1 and 2 (IDH1/2), represents a significant advancement in the management of IDH-mutant gliomas. This review explores the clinical implications of its recent FDA approval for grade 2 IDH-mutant astrocytomas and oligodendrogliomas. We delve into the pivotal INDIGO trial, which demonstrated substantial improvements in progression-free survival, and discuss vorasidenib’s pharmacokinetics, safety profile, and dosing guidelines. Additionally, we analyze its role within evolving treatment paradigms, including watchful waiting, IDH-targeted therapy, and integration with radiotherapy and chemotherapy. Comparative insights into traditional and novel approaches highlight the potential of vorasidenib to delay invasive therapies while preserving quality of life. Challenges such as adverse effects, long-term safety, and its application to higher-grade gliomas are also addressed. This comprehensive review underscores the transformative impact of vorasidenib and emphasizes the necessity of multidisciplinary care and patient-centered decision-making in glioma management.

## 1 Introduction to Vorasidenib

Grade 2 gliomas, encompassing IDH-mutant astrocytomas and oligodendrogliomas, are characterized by distinct clinical, radiological, and molecular features. Clinically, they often present in younger adults (20–40 years) with seizures, focal neurological deficits, or incidental findings on imaging. Radiologically, they typically appear as non-enhancing or minimally enhancing T2/FLAIR hyperintense lesions on MRI, with oligodendrogliomas more commonly located in the frontal lobes and astrocytomas showing a broader distribution. Molecularly, IDH1/2 mutations are a hallmark, with oligodendrogliomas defined by 1p/19q codeletion and astrocytomas often harboring ATRX and TP53 mutations (1). These features underscore the importance of molecular diagnostics in guiding treatment decisions for these tumors.

Vorasidenib represents a first-in-class, brain-penetrant, orally bioavailable inhibitor targeting mutant isocitrate dehydrogenase 1 and 2 (IDH1/2) enzymes. It garnered approval from the United States Food and Drug Administration (FDA) in August 2024 for the treatment of adult and pediatric patients aged 12 years and older diagnosed with World Health Organization (WHO) grade 2 astrocytoma or oligodendroglioma harboring IDH mutations (2). It is currently under review by other health authorities, including the European Medicines Agency (EMA). While its indication remains for grade 2 IDH-mutant gliomas, specific aspects, such as prescription restrictions for patients with residual tumor, may vary (2). IDH1/2 mutations play a pivotal role in oncogenesis by catalyzing the production of the oncometabolite 2-hydroxyglutarate (2-HG). This metabolite disrupts normal cellular differentiation and promotes tumorigenesis through the induction of epigenetic alterations (3). Vorasidenib exerts its therapeutic effect by selectively inhibiting mutant IDH1/2, thereby reducing intracellular 2-HG levels and providing a targeted therapeutic strategy for patients with limited treatment options beyond conventional surgery and radiotherapy (4).

Its efficacy was demonstrated in the pivotal INDIGO trial (NCT04164901), a phase III randomized controlled trial, which showed that Vorasidenib significantly prolonged progression-free survival (PFS) in patients with grade 2 IDH-mutant gliomas, from 11.1 months to 27.7 months (hazard ratio [HR], 0.39; 95% confidence interval [CI], 0.27-0.56) (2). The trial enrolled patients who had undergone surgery but not radiotherapy or chemotherapy, excluding those with uncontrolled symptoms such as refractory seizures to ensure clinical stability. Additionally, Vorasidenib had a low incidence of fatigue (comparable to placebo), suggesting minimal impact on quality of life (QoL), and preliminary data hint at potential seizure control benefits due to 2-HG reduction, though further studies are needed (2).

The development of Vorasidenib was preceded by phase 0 and 1 trials, which established its safety, pharmacokinetics, and preliminary efficacy in IDH-mutant gliomas. In a phase 1 trial (NCT02481154), Vorasidenib demonstrated a favorable safety profile and reduced 2-HG levels in enhancing tumors, supporting its further evaluation (5). Other IDH inhibitors, such as ivosidenib, have been approved for IDH1-mutant acute myeloid leukemia but have limited data in gliomas, highlighting Vorasidenib’s unique role in this context (6).

For both IDH-mutant grade 2 astrocytomas and oligodendrogliomas, maximal safe resection remains the cornerstone of initial management, with supramarginal resection (SMR) enhancing the feasibility of observation by reducing residual tumor burden and delaying progression, as supported by recent studies (7). Postoperative surveillance typically involves contrast-enhanced MRI every 3 to 4 months for the first 1–2 years in low-risk patients managed with observation, with intervals extended thereafter based on tumor stability (8). This review synthesizes current evidence on the management of IDH-mutant grade 2 astrocytomas and oligodendrogliomas, focusing on risk stratification, treatment strategies, and emerging therapeutic options.

### 1.1 Pretreatment counseling

Prior to initiating vorasidenib therapy, comprehensive patient counseling is essential. Preclinical investigations have demonstrated that vorasidenib carries a risk of fetal harm; however, human data regarding its use during pregnancy are presently lacking (2). Patients must be thoroughly informed of this potential risk and strongly advised to utilize effective non-hormonal contraception throughout the treatment period and for three months after the final administration. It is also important to note that vorasidenib may decrease plasma concentrations of hormonal contraceptives, thereby elevating the risk of contraceptive failure and breakthrough bleeding (2). Furthermore, animal studies have indicated potential impairment of fertility in both male and female subjects (2). These reproductive risks underscore the critical importance of thorough patient education and the acquisition of informed consent to ensure well-considered treatment decisions.

### 1.2 Dosing and administration

The posology of vorasidenib is weight-adjusted for pediatric patients and follows a standardized regimen for adults. The recommended oral dose is 40 mg once daily for adults and children weighing 40 kg or more, and 20 mg once daily for children weighing less than 40 kg (2). The medication can be administered at any time of day, irrespective of food intake. Patients should be instructed to swallow the tablets whole; splitting, crushing, or chewing is contraindicated. Routine antiemetic prophylaxis is generally not warranted due to the low incidence of associated nausea and vomiting.

Pharmacokinetic Properties: Vorasidenib undergoes extensive hepatic metabolism primarily mediated by cytochrome P450 1A2 (CYP1A2) enzymes (2). Therefore, the concurrent administration of strong CYP1A2 inhibitors (e.g., ciprofloxacin) or potent inducers (e.g., phenytoin) should be avoided, as these agents have the potential to significantly alter vorasidenib plasma concentrations, potentially impacting therapeutic efficacy or increasing the risk of adverse events (2).

### 1.3 Monitoring

Given the potential for hepatotoxicity, meticulous monitoring of hepatic function is paramount throughout vorasidenib therapy. Baseline liver function tests, encompassing alanine aminotransferase (ALT), aspartate aminotransferase (AST), gamma-glutamyl transferase (GGT), total bilirubin, and alkaline phosphatase, should be performed before commencing treatment. These parameters should be serially assessed every two weeks during the initial two months, followed by monthly evaluations for the subsequent two years, and thereafter as clinically indicated (2). Specific dose modifications in response to hepatotoxicity are detailed within the official prescribing information (2).

Furthermore, baseline renal function, electrolyte levels, and a complete blood count (CBC) should be obtained, with subsequent monthly monitoring to detect any emergent abnormalities (2).

### 1.4 Adverse effects

Hepatotoxicity stands out as the most clinically significant adverse effect associated with vorasidenib, warranting diligent monitoring. Data from the pivotal INDIGO trial indicated that elevations in ALT and AST of any grade were observed in 59% and 46% of patients, respectively, with grade 3 or 4 elevations reported in 10% and 5% (2, 9). The median time to the initial elevation of these liver enzymes was 57 days. Further liver-related abnormalities included elevations in GGT (34%), alkaline phosphatase (9%), and bilirubin (5%) (2). Notably, severe events such as two cases of autoimmune hepatitis and hepatic failure were reported, highlighting the potential for significant hepatic injury (2).

Hematologic adverse effects were generally of low grade, encompassing increased hemoglobin (13%, no grade 3/4), lymphopenia (11%, 1.8% grade 3/4), neutropenia (14%, 2.4% grade 3/4), and thrombocytopenia (12%, no grade 3/4) (2).

Other frequently reported adverse effects (occurring in >15% of patients) were predominantly low-grade, including musculoskeletal pain, seizure, diarrhea, and COVID-19 infection (2). The use of vorasidenib in clinical practice is still evolving, and many considerations presented in this review reflect the authors’ perspectives, as a broad consensus within the scientific community on its use beyond current prescription labels has yet to be established. Vorasidenib has been incorporated into the recent ASCO-SNO glioma guideline update (2025) (10, 11), but it has not yet been included in other societal guidelines, such as those by the European Association of Neuro-Oncology (EANO) (12).

## 2 Surveillance strategies for IDH-mutant grade 2 gliomas

Postoperative surveillance for IDH-mutant grade 2 gliomas typically involves serial contrast-enhanced MRI scans to monitor local recurrence at resection margins. Subtle changes in non-enhancing T2/FLAIR signal intensity can be challenging to interpret and may require comparison with the earliest postoperative imaging as a baseline. Establishing definitive tumor progression often takes several years, and not all minor changes warrant immediate intervention, particularly in slow-growing lesions, making the timing of further treatment a complex decision. Amino acid PET (e.g., [18F]FET-PET or [11C]MET-PET) enhances monitoring by distinguishing postoperative scarring or inflammation from tumor recurrence (specificity ~85-90%) and detecting metabolic changes 3–6 months before anatomical changes on MRI, aiding early identification of progression (13, 14).

## 3 Grade 2 oligodendroglioma

Oligodendroglial tumors, comprising 5-10% of glial neoplasms, typically arise in the fourth to sixth decades, with grade 2 tumors presenting earlier than grade 3 (1). Despite a protracted course, they are linked to limited survival. Historically, treatment relied on tumor grade and trials including both astrocytic and oligodendroglial tumors, predating recognition of their distinct molecular profiles. Retrospective 1p/19q status assessments later refined treatment per the WHO classification (1). Per WHO criteria, oligodendroglioma diagnosis requires IDH mutation and 1p/19q co-deletion (5). Though histopathologically graded as 2 or 3, modern molecular diagnostics have nuanced their biological and prognostic differences, particularly for radiotherapy and chemotherapy, enhancing treatment precision beyond traditional grading

For patients with newly diagnosed grade 2 oligodendroglioma, postoperative management strategies, outside the context of clinical trials, typically encompass watchful waiting, radiotherapy (RT) combined with chemotherapy, and IDH-targeted therapy. The adoption of an IDH inhibitor in this clinical setting is primarily supported by the findings of the pivotal INDIGO trial, which evaluated vorasidenib, a dual inhibitor of IDH1 and IDH2. Notably, vorasidenib received approval from the United States Food and Drug Administration (FDA) in August 2024 for the treatment of grade 2 oligodendroglioma and grade 2 IDH-mutant astrocytoma (2).

### 3.1 Risk stratification

The management of patients with grade 2 IDH-mutant, 1p/19q-codeleted oligodendrogliomas is evolving with the introduction of IDH-targeted therapies and advanced risk stratification frameworks. Traditionally, treatment decisions for immediate postoperative radiotherapy and chemotherapy have relied on factors such as age, extent of resection, tumor size, and histological grade. However, with integrated molecular diagnostics, these factors are increasingly viewed as insufficient on their own. For example, while age (>40 years) was once a key high-risk indicator, recent evidence suggests that molecular profiles may better predict outcomes, with some younger patients showing high-risk features necessitating earlier intervention (12). Similarly, histological grade alone does not fully reflect the clinical heterogeneity of grade 2 oligodendrogliomas, as molecular characteristics can drive divergent behaviors despite similar grades (15). Notably, emerging molecular markers like CDKN2A/B homozygous deletion are linked to poorer prognosis in IDH-mutant gliomas, refining risk assessment and guiding decisions, such as the use of vorasidenib (16).

Current risk stratification prioritizes high-risk features that justify early intervention, including uncontrolled symptoms (e.g., refractory seizures), contrast enhancement on MRI, and significant residual tumor volume (>1 cm) (12). For oligodendrogliomas, which typically grow slowly (annual diameter increase of 4–6 mm), volumetric analysis (see General Management Principles) enhances risk evaluation by distinguishing indolent tumors suitable for observation or IDH-targeted therapies from those requiring aggressive treatment (17). This refined approach broadens the group of patients eligible for initial watchful waiting or IDH-targeted therapies, potentially delaying or avoiding immediate radiotherapy and chemotherapy (12).

### 3.2 Patients following gross total resection

Watchful waiting has been the conventional postoperative management strategy for the majority of patients with grade 2 oligodendroglioma after achieving a gross total resection. This approach is predicated on their typically low risk of early clinical progression, potentially obviating the need for immediate adjuvant therapy for an extended period. While IDH-targeted therapy presents a potential avenue for prolonging progression-free survival in this cohort, it is important to note that patients without measurable residual disease were not enrolled in the pivotal INDIGO trial. Furthermore, long-term safety data for prolonged vorasidenib administration are currently limited to a small number of patients who participated in phase 1 clinical trials. Consequently, this remains an area of clinical uncertainty, underscoring the importance of individualized treatment decisions informed by shared decision-making between the clinician and the patient.

For patients who opt for watchful waiting as their initial management strategy, the supporting rationale, surveillance protocols, and management strategies at the time of disease progression are outlined below:

#### 3.2.1 Rationale for observation

Maximal safe resection, often enhanced by supramarginal resection (SMR), remains the cornerstone of initial management for grade 2 IDH-mutant, 1p/19q-codeleted oligodendrogliomas, as discussed in Section 1. For low-risk patients—defined by younger age (<40 years), gross total resection (GTR), and absence of uncontrolled symptoms—observation following surgery is a reasonable strategy, given the slower growth rate of these tumors (typically 4–6 mm/year) (13). This approach delays the need for radiotherapy (RT) and chemotherapy, minimizing potential long-term toxicities such as cognitive decline or secondary malignancies.

#### 3.2.2 Surveillance interval

Following resection of grade 2 oligodendrogliomas (IDH-mutant, 1p/19q codeleted), patients on observation undergo MRI scans with intervals extended to 6–9 months after 1–2 years of stability, as outlined in Section 2. Due to their slower growth, amino acid PET signals may be weaker than in astrocytomas, yet this modality still supports precise monitoring and timely intervention when needed.

#### 3.2.3 Management at the time of progression

Management at the Time of Progression: The management of recurrent or progressive tumor in patients who have undergone an initial period of observation following resection is highly individualized. Treatment decisions are guided by several factors, including the rate of change in tumor size between consecutive MRI scans, the overall tumor volume, its anatomical location within the brain and feasibility of surgical resection, the patient’s preferences regarding further surgical intervention, the presence or absence of contrast enhancement, and the histopathological grade of the progressive tumor, if available.

Common clinical scenarios encountered in this setting include:

① For patients with recurrent tumors where repeat resection is deemed safe and likely to achieve extensive or gross total removal, surgical re-resection represents a viable option. If the subsequent resection results in minimal or no residual tumor and pathological analysis confirms a grade 2 tumor, a watch-and-wait strategy may again be a suitable consideration.
② In patients presenting with a relatively low volume of recurrent, non-enhancing tumor, or in those who are not candidates for or decline further surgical intervention, vorasidenib may represent a reasonable next-line therapeutic option, mirroring its potential use in newly diagnosed patients with residual disease.
③ For patients experiencing a large or rapidly progressive recurrence, repeat surgical resection is often indicated for tumor debulking and to obtain tissue for confirming the diagnosis and histological grade. Subsequent postoperative management decisions are contingent upon the extent of resection achieved and the pathological characteristics of the recurrent tumor, as detailed in the section addressing newly diagnosed patients.

### 3.3 Patients with uncontrolled symptoms or progression on Vorasidenib

“Uncontrolled disease-related symptoms” in the context of grade 2 IDH-mutant, 1p/19q-codeleted oligodendrogliomas typically refer to persistent neurological manifestations that significantly impact quality of life, such as refractory seizures, focal neurological deficits (e.g., limb weakness or sensory loss), cognitive impairment (e.g., memory decline), or symptoms of increased intracranial pressure (e.g., headache, nausea). Among these, refractory seizures are particularly prevalent, affecting up to 70-90% of patients with low-grade gliomas due to the epileptogenic effects of the IDH mutation-driven oncometabolite 2-hydroxyglutarate (2-HG). For the majority of patients experiencing uncontrolled disease-related symptoms or demonstrating disease progression following initial treatment with vorasidenib, postoperative therapy with radiotherapy and chemotherapy is recommended. An exception to this approach may be considered if repeat surgical resection is deemed safe and likely to provide clinical benefit before the initiation of subsequent lines of therapy. The INDIGO trial excluded patients with uncontrolled symptoms, including those with refractory seizures (2). Preclinical studies suggest that IDH inhibitors like vorasidenib, by reducing 2-HG levels, may decrease epileptogenic activity, as 2-HG has been shown to mimic glutamate and enhance neuronal excitability (18). However, the potential benefit of vorasidenib for seizure control remains to be clinically validated, particularly in patients with refractory seizures, necessitating further research.

The evidence supporting the use of combined radiotherapy and chemotherapy in low-grade oligodendrogliomas is primarily derived from the Radiation Therapy Oncology Group (RTOG) 9802 trial. This study randomized 251 patients with a supratentorial low-grade glioma to receive postoperative radiotherapy (54 Gy delivered in 30 fractions) with or without six cycles of adjuvant PCV chemotherapy (19). Eligibility criteria included patients aged 18 to 39 years with subtotal resection or biopsy, or those aged 40 years or older with any extent of resection. The study population comprised patients with diffuse astrocytoma (26%), oligodendroglioma (42%), and mixed astrocytoma/oligodendroglioma (32%). Randomization was stratified based on age, presence of contrast enhancement, and predominant histology (astrocytic versus oligodendroglial). At a median follow-up of 11.9 years, the median overall survival was significantly longer in the group receiving postoperative radiotherapy followed by PCV chemotherapy compared to the radiotherapy-alone group (13.3 versus 7.8 years; hazard ratio [HR], 0.59; p = 0.03) (20). The incidence of grade 3 and 4 hematologic toxicity was 8% and 3% in the radiotherapy-alone arm, respectively, compared to 51% and 15% in the radiotherapy-plus-PCV arm. Notably, there were no treatment-related deaths and no reported cases of secondary leukemia in this trial (19). While the survival benefit associated with PCV was observed across all histological subtypes, the most pronounced effect was evident in patients with histologically confirmed oligodendroglioma (n = 101; hazard ratio [HR], 0.43; 95% confidence interval [CI], 0.23-0.82) (20). Subsequent *post hoc* molecular analysis of the RTOG 9802 trial, conducted on 42% of enrolled cases, further highlighted a significant survival advantage in patients with molecularly confirmed IDH-mutant, 1p/19q-codeleted oligodendrogliomas (hazard ratio [HR], 0.21; p = 0.029) (21).

In addition to the findings from the RTOG 9802 trial, preliminary data from the ECOG-ACRIN E3F05 trial, presented in abstract format, suggest that radiotherapy combined with concurrent and adjuvant temozolomide (TMZ) is also a viable treatment option for low-grade glioma, including 1p/19q-codeleted oligodendroglioma (22). Recent evidence has provided insights into the comparison of TMZ with PCV in IDH-mutant, 1p/19q-codeleted gliomas. A POLA network study by Kacimi SEO, Dehais C, Feuvret L, Chinot O, Carpentier C, Bronnimann C compared RT plus PCV versus RT plus TMZ in grade 3 oligodendrogliomas, reporting a significant overall survival (OS) benefit with PCV (5-year OS: 89% vs. 75%; 10-year OS: 72% vs. 60%; HR 0.53, 95% CI: 0.30-0.92, p=0.025) (23). Although this study focused on grade 3 tumors, it suggests that PCV may offer superior survival outcomes compared to TMZ in IDH-mutant, 1p/19q-codeleted gliomas, potentially informing treatment decisions for grade 2 oligodendrogliomas as well. However, TMZ offers advantages over PCV in terms of ease of administration, improved patient tolerability, and more consistent availability in certain geographical regions. The ongoing “CODEL” trial, a randomized study in patients with 1p/19q-codeleted tumors, is further comparing radiotherapy plus PCV with radiotherapy plus concurrent and adjuvant temozolomide to clarify their relative efficacy. The authors note that the recommendation for radiotherapy and chemotherapy as the preferred approach in this context reflects their perspective, acknowledging that clinical practice may vary based on patient-specific factors and emerging evidence, as a broad consensus within the scientific community on the use of vorasidenib beyond current prescription labels has yet to be established.

## 4 Grade 2 astrocytoma

Surgery alone is not curative for patients with diffuse gliomas, including grade 2 tumors, and nearly all patients ultimately require additional anticancer therapy. However, the optimal timing of such therapy in patients with grade 2 tumors remains uncertain, and decisions regarding immediate versus delayed postoperative treatment should be tailored to individual circumstances (16, 24,11, 25, 26).

Following a new diagnosis of IDH-mutant grade 2 astrocytoma, patients have three primary options for postoperative management outside clinical trials: watchful waiting, radiotherapy (RT) combined with chemotherapy, or IDH-targeted therapy. The use of an IDH inhibitor in this context is supported by the INDIGO trial, which evaluated vorasidenib, an IDH1/2 inhibitor approved by the US Food and Drug Administration (FDA) in August 2024 for grade 2 IDH-mutant astrocytoma and oligodendroglioma.

### 4.1 Risk stratification

The management of patients with grade 2 IDH-mutant astrocytomas (non-1p/19q codeleted) is evolving as emerging data on IDH-targeted therapies refine risk assessment and challenge traditional treatment paradigms. Historically, risk stratification has relied on factors such as age, extent of resection, tumor size, and histological grade to guide decisions about immediate postoperative radiotherapy (RT) and chemotherapy. However, in the era of integrated molecular diagnostics, these factors are increasingly viewed as insufficient on their own. For instance, while age (>40 years) was once considered a high-risk factor, recent studies suggest that molecular features may outweigh age in predicting outcomes, with younger patients sometimes harboring aggressive molecular profiles that require earlier intervention (12). Similarly, histological grade alone does not fully capture the biological behavior of grade 2 astrocytomas, as molecular characteristics can drive divergent progression rates despite similar grades (15). Notably, emerging molecular markers like CDKN2A/B homozygous deletion are associated with increased risk of progression and malignant transformation, influencing decisions regarding therapies such as vorasidenib (15).

Current risk stratification continues to acknowledge high-risk features that may warrant early RT and chemotherapy, including uncontrolled symptoms, contrast enhancement on MRI, and significant residual tumor volume (12). For astrocytomas, which typically exhibit faster growth (annual diameter increase of 8–10 mm) compared to oligodendrogliomas (4–6 mm/year), volumetric analysis (see General Management Principles) is particularly valuable for capturing their aggressive behavior and identifying patients who may require immediate RT and chemotherapy versus those suitable for watchful waiting or IDH-targeted therapies like vorasidenib (17). This refined framework also broadens eligibility for delaying RT and chemotherapy in select patients (12).

### 4.2 Gross total resection

Watchful waiting has traditionally been the preferred approach for most patients with grade 2 IDH-mutant astrocytoma following gross total resection, given their low risk of early clinical progression and the potential to delay additional therapy for years. IDH-targeted therapy, such as vorasidenib, offers an alternative to extend progression-free survival. However, the INDIGO trial excluded patients without measurable disease, and safety data for prolonged vorasidenib use are limited to a small cohort from phase 1 trials. This uncertainty underscores the need for individualized decisions, guided by shared decision-making.

For patients opting for watchful waiting, the anticipated natural history, surveillance strategy, and management at progression are outlined below:

#### 4.2.1 Natural history after complete resection

Grade 2 IDH-mutant astrocytomas (non-1p/19q codeleted) exhibit a more aggressive natural history compared to oligodendrogliomas, with a higher risk of malignant transformation over time. Maximal safe resection, often enhanced by supramarginal resection (SMR), remains the cornerstone of initial management, as discussed in Section 1. However, even with maximal resection, these tumors often recur due to their infiltrative nature and faster growth rate (typically 8–10 mm/year). Observation may be considered for low-risk patients following surgery, but the potential for progression necessitates careful risk stratification and vigilant monitoring.

#### 4.2.2 Surveillance interval

Grade 2 astrocytomas (IDH-mutant, non-1p/19q codeleted) require frequent monitoring due to their higher risk of progression and malignant transformation. For low-risk patients on observation, MRI scans occur every 3–4 months for the first 2 years, then every 6 months, as per Section 2. The higher metabolic activity of astrocytomas enhances the utility of amino acid PET in detecting early progression. Rapid tumor regrowth between consecutive MRIs, along with new neurologic symptoms, contrast enhancement, or peritumoral edema, may indicate transformation to a higher-grade tumor.

#### 4.2.3 Management at the time of progression

Management of recurrent or progressive tumors in patients observed after initial resection of an IDH-mutant grade 2 glioma is tailored to the individual. Treatment decisions consider factors such as the rate of tumor growth between consecutive magnetic resonance imaging (MRI) scans, overall tumor volume, location and resectability, patient preferences regarding further surgery, the emergence of contrast enhancement, and, when available, the histopathologic grade of the progressive tumor.

Common management scenarios for recurrent tumors include the following:

① In patients with recurrent tumors where re-resection is deemed safe and likely to achieve extensive or gross total removal, re-resection is a viable option. If minimal or no residual tumor remains and pathology confirms a grade 2 tumor, a watch-and-wait approach may again be appropriate.
② For patients with a relatively small volume of recurrent, nonenhancing tumor, or those ineligible for or declining further surgery, vorasidenib may be a reasonable next-line therapy, similar to its use in newly diagnosed patients with residual disease. (See ‘Subtotal resection, no uncontrolled symptoms’ below.)
③ In cases of large or rapidly progressive recurrence, re-resection is often indicated for debulking and may also clarify tumor grade and molecular status if not previously established. Postoperative management hinges on the extent of resection and the histopathologic findings, as outlined below for newly diagnosed patients.

### 4.3 Subtotal resection, no uncontrolled symptoms

For most patients with residual disease after surgery and no uncontrolled disease-related symptoms, we recommend IDH-targeted therapy with vorasidenib over radiotherapy (RT) plus chemotherapy or watchful waiting. This strategy prioritizes vorasidenib’s potential to delay RT and chemotherapy, though key uncertainties persist, including its impact on overall survival and quality of life.

Notably, while the INDIGO trial excluded patients with enhancing disease, we do not view this as an absolute contraindication to vorasidenib in otherwise stable patients without uncontrolled symptoms, provided the risk of an under-graded tumor is minimal (e.g., small biopsy specimen or discrepancy between tumor grade and clinical presentation). Most newly diagnosed grade 2 IDH-mutant astrocytomas exhibit no or minimal enhancement. Although data from other IDH inhibitor trials indicate lower response rates in enhancing versus nonenhancing recurrent or progressive tumors, these findings may not apply to newly diagnosed grade 2 tumors. No consensus exists on residual tumor size as a criterion for selecting next-line therapy, requiring clinicians to exercise judgment in identifying patients suitable for vorasidenib to safely defer RT and chemotherapy. Multidisciplinary discussion is advised.

The efficacy of vorasidenib is supported by the multicenter phase 3 INDIGO trial, which randomized 331 patients aged 12 years or older with residual or recurrent grade 2 IDH-mutant glioma (9). Eligible patients had undergone no prior therapy beyond surgery, had measurable nonenhancing disease (≥1 cm in two dimensions), a Karnofsky Performance Status of ≥80, were not receiving glucocorticoids, and were deemed suitable for watchful waiting. Participants received either oral vorasidenib (40 mg daily) or placebo. The median age was approximately 40 years (range, 16-71), with over 80% having residual disease >2 cm in diameter, meeting historical ‘high-risk’ criteria for low-grade glioma (e.g., per National Comprehensive Cancer Network [NCCN] guidelines (27)). Histologically, 52% had oligodendroglioma and 48% had astrocytoma.

At a median follow-up of 14.2 months, vorasidenib significantly prolonged progression-free survival compared with placebo (27.7 vs. 11.1 months; hazard ratio [HR], 0.39; 95% CI, 0.27-0.56) (9). The probability of avoiding subsequent treatment was higher with vorasidenib at 18 months (85.6% vs. 47.4%) and 24 months (83.4% vs. 27.0%). Data on seizure control, quality of life, and overall survival remain pending. Vorasidenib was generally well tolerated, with serious adverse events occurring in <2% of patients. The most frequent grade 3 or higher adverse event was elevated alanine aminotransferase (9.6%). Treatment discontinuation rates were <4% in both arms.

Several IDH inhibitors, approved by the FDA for other malignancies, are under investigation for IDH-mutant gliomas, including ivosidenib, olutasidenib, and safusidenib (mutant IDH1 inhibitors) and enasidenib (mutant IDH2 inhibitor). Additional data from well-designed trials are required to define their role in the management of IDH-mutant gliomas.

### 4.4 Uncontrolled symptoms or progression on Vorasidenib

Postoperative radiotherapy (RT) and chemotherapy are recommended for most patients with uncontrolled disease-related symptoms or those who progress on initial vorasidenib therapy, unless repeat surgery is deemed safe and clinically beneficial before proceeding to the next line of treatment. The authors note that this recommendation reflects their perspective, acknowledging that clinical practice may vary based on patient-specific factors and emerging evidence, as a broad consensus within the scientific community on the use of vorasidenib beyond current prescription labels has yet to be established.

#### 4.4.1 Rationale for RT plus chemotherapy

In patients with IDH-mutant grade 2 astrocytoma, evidence for an overall survival benefit from radiotherapy (RT) plus chemotherapy (PCV or temozolomide) over RT alone stems from multiple trials. The RTOG 9802 trial, involving 251 high-risk low-grade glioma patients, compared postoperative RT (54 Gy) alone to RT with six cycles of PCV (19, 20, 22, 28–30). High-risk criteria included ages 18–39 with subtotal resection/biopsy or ≥40 with any extent of resection. Histologies were astrocytoma (26%), oligodendroglioma (42%), and mixed (32%). At 11.9 years median follow-up, RT plus PCV significantly extended median overall survival (13.3 vs. 7.8 years; HR, 0.59; P=0.003) and progression-free survival (20). Toxicities were higher with PCV (grade 3: 51%, grade 4: 15%) than RT alone (grade 3: 8%, grade 4: 3%), with no treatment-related deaths (19). MMSE scores improved in both arms, unaffected by PCV (31). Survival benefits persisted across histologies, notably in molecular subgroups like IDH-mutant astrocytoma (HR, 0.38; P=0.013) and oligodendroglioma (HR, 0.21; P=0.029), despite limited sample sizes (21). The CATNON trial (grade 3 tumors) and ECOG-ACRIN E3F05 trial (low-grade gliomas) further support RT plus temozolomide (11, 13, 16). Chemotherapy alone is generally not advised due to lower chemosensitivity, except in cases with large RT fields, though progression-free survival may be shorter (32).

#### 4.4.2 Choice of PCV versus temozolomide

In IDH-mutant grade 2 astrocytomas, both PCV and temozolomide are viable adjuvant therapies with radiotherapy (RT), though no direct comparative trials exist. Temozolomide is increasingly favored due to its efficacy in grade 2 and 3 tumors, ease of administration, better tolerability, and availability, supported by the CATNON trial (19). Conversely, PCV’s benefit is evidenced by the RTOG 9802 trial, showing improved survival with RT plus PCV over RT alone in low-grade gliomas (33–35). For temozolomide, we typically use 12 adjuvant cycles post-RT, omitting concurrent dosing during RT, as CATNON found no additional benefit from concurrent temozolomide in grade 3 tumors. The ECOG-ACRIN E3F05 trial included both concurrent and adjuvant temozolomide but couldn’t isolate their effects (22). The RTOG 0424 study, a single-arm trial with 129 high-risk low-grade glioma patients (≥3 risk factors: age ≥40, astrocytoma, bihemispheric tumor, >6 cm diameter, neurologic deficit), used RT (54 Gy) with concurrent temozolomide, followed by monthly cycles (33–35). At 9 years median follow-up, 5-year progression-free survival was 47%, overall survival was 61%, median survival was 8.2 years, and 10-year survival was 34%, outperforming RT-alone historical controls (36, 37). Grade 3 and 4 hematologic toxicities occurred in 44% and 10%, respectively.

### 4.5 During Vorasidenib therapy

We obtain a contrast-enhanced brain MRI at baseline and every 3 months for the first 3 years of therapy. For patients stable on vorasidenib beyond this period, imaging intervals may be extended to every 6 months if no new symptoms emerge.

## 5 Grade 3 and grade 4 gliomas

Vorasidenib, an oral dual IDH1/IDH2 inhibitor, received FDA approval in August 2024 for IDH-mutant grade 2 gliomas, based on the phase 3 INDIGO trial (NCT04164901). This trial demonstrated a significant progression-free survival (PFS) benefit (27.7 months vs. 11.1 months with placebo; HR, 0.39) (9), though it was limited to grade 2 tumors. Its application in grade 3 (e.g., IDH-mutant astrocytoma) and grade 4 (e.g., glioblastoma) gliomas remains unapproved and underexplored. For grade 3 gliomas, a phase 1 study (NCT02481154) of vorasidenib in recurrent IDH-mutant gliomas, including a small grade 3 subset, reported a median PFS of 36.8 months for non-enhancing tumors but only 3.6 months for enhancing tumors—common in grade 3—suggesting limited efficacy in aggressive cases (5). Another phase 1 trial (NCT03343197) showed a 92.6% reduction in intratumoral 2-hydroxyglutarate, though grade 3-specific outcomes were not isolated (38). In grade 4 gliomas, evidence is scarcer; the NCT02481154 trial’s short PFS (3.6 months) in enhancing tumors indicates poor single-agent control, likely due to rapid progression and molecular complexity (33). An ongoing trial (NCT05669339) combines vorasidenib with pembrolizumab for recurrent IDH1-mutant gliomas, including grade 4 cases, but data are pending (39). Recent studies have further explored vorasidenib’s potential in broader patient populations. A 2025 review highlighted its approval by both the FDA and the Australian TGA for patients aged 12 years and older with WHO grade 2 gliomas (IDH1 or IDH2 mutant), reaffirming its PFS benefit (27.7 months vs. 11.1 months) and good tolerability in children and adolescents, with grade 3 or higher adverse events like transaminase elevation occurring in less than 50% of cases (40). While these data focus on grade 2 gliomas, the authors suggest future research should evaluate vorasidenib’s role in higher-grade gliomas in pediatric populations, particularly in combination therapies. Additionally, another 2025 review raised critical questions about vorasidenib’s broader applicability, including its efficacy in WHO grade 3 or 4 gliomas, optimal timing for initiation and cessation of treatment, and potential integration with radiotherapy or chemotherapy (41). The authors also noted vorasidenib’s possible benefits in seizure control and quality of life, though these outcomes require validation in larger trials. These insights underscore the need for further studies to explore combination strategies and define vorasidenib’s role across all glioma grades.

## 6 Neurocognition, quality of life, and fertility preservation in low-grade gliomas

Low-grade gliomas (LGGs), including IDH-mutant grade 2 astrocytomas and oligodendrogliomas, often affect younger adults, making neurocognitive function, quality of life (QoL), return-to-work capability, and fertility preservation critical considerations in their management. LGGs can impair cognitive domains such as memory, attention, and executive function, particularly when located in eloquent brain regions. Studies indicate that up to 50% of LGG patients experience cognitive deficits at diagnosis, exacerbated by tumor growth, seizures, or treatment effects (42). Radiotherapy (RT), while effective, may contribute to long-term cognitive decline, especially in patients receiving higher doses (>50 Gy) or whole-brain RT. The RTOG 9802 trial suggests that carefully dosed RT (54 Gy) may mitigate cognitive impact, though detailed neurocognitive assessments are limited (5). However, temozolomide (TMZ) and PCV can cause fatigue and hematologic toxicities, further affecting QoL.

Vorasidenib, as an IDH-targeted therapy, may offer cognitive and QoL advantages by delaying RT and chemotherapy. The INDIGO trial reported a low incidence of fatigue with vorasidenib (comparable to placebo), suggesting minimal QoL disruption (Mellinghoff et al., 2023). Preliminary data also hint at potential seizure control benefits due to 2-HG reduction, which could improve cognitive function and daily living, though mature data are pending. Return-to-work rates in LGG patients vary widely (30-70%), influenced by tumor location, seizure control, and treatment side effects. Vocational rehabilitation and cognitive training may support reintegration, particularly for patients on observation or vorasidenib.

Fertility preservation is a key concern for younger LGG patients. RT to the hypothalamic-pituitary axis may impair fertility, with studies reporting a 20-30% risk of hypogonadism in LGG patients post-RT (43). Chemotherapy regimens like PCV and TMZ, as alkylating agents, carry risks of ovarian or testicular dysfunction, particularly with lomustine. Vorasidenib’s prescribing information highlights potential fertility impairment in animal studies and advises non-hormonal contraception due to reduced efficacy of hormonal contraceptives (FDA, 2024). Patients should be counseled on fertility preservation options, such as sperm or egg banking, before initiating RT, chemotherapy, or vorasidenib. Multidisciplinary care involving neuropsychologists, social workers, and fertility specialists is essential to optimize cognitive outcomes, QoL, and reproductive planning in LGG management.

## 7 Decision-making algorithm for IDH-mutant gliomas

**Figure 1:**
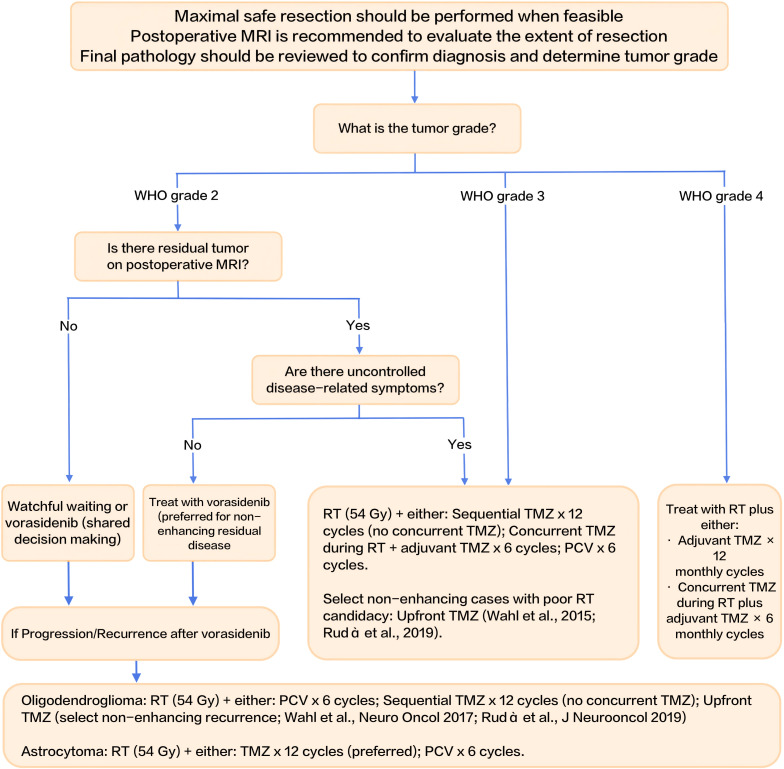
Decision-making algorithm for postoperative management of IDH-mutant gliomas.

To consolidate the management strategies discussed throughout this review, we present a decision-making algorithm for the postoperative management of IDH-mutant gliomas in **Figure 1**. This flowchart integrates key clinical factors—WHO tumor grade, presence of residual tumor on MRI, and uncontrolled disease-related symptoms—to guide treatment decisions. It outlines pathways for watchful waiting, IDH-targeted therapies like vorasidenib, and more intensive treatments such as radiotherapy (RT) with chemotherapy (TMZ or PCV). The algorithm serves as a practical tool for clinicians, facilitating individualized treatment planning based on patient-specific risk profiles and tumor characteristics.

This flowchart summarizes the postoperative management strategies for IDH-mutant gliomas, integrating risk stratification, tumor grade, presence of residual tumor on MRI, and uncontrolled disease-related symptoms to guide treatment decisions. RT: radiotherapy; TMZ: temozolomide; PCV: procarbazine, lomustine, and vincristine.

To further summarize and differentiate the clinical management strategies for grade 2 IDH-mutant gliomas, **Tables 1** and **2** outline the treatment paradigms for oligodendrogliomas and astrocytomas, respectively, highlighting their distinct risk profiles, preferred treatment options, and applicable clinical scenarios.

**Table 1:** Clinical management paradigm for grade 2 oligodendrogliomas (IDH-mutant, 1p/19q codeleted).

Risk Level	Risk Features	Treatment Options	Applicable Scenarios
Low Risk	Age<40 years, gross total resection(GTR), no uncontrolled symptoms, slow growth rate (<4-6 mm/year, volume growth<10%or<8 cm³/year), typically non-enhancing	Watchful waiting Vorasidenib (preferred) (2)	No uncontrolled symptoms (e.g., refractory seizures),non-enhancing residual disease (<1 cm), slower tumor growth allows for delayed RT/chemotherapy
High Risk	Uncontrolled symptoms(e.g., refractory seizures), contrast enhancement on MRI, significant residual tumor(>1cm),rapid growth (>10%or>8 cm³/year)	RT(54 Gy)+PCV (preferred) (20, 23) RT(54 Gy)+ sequential TMZ (13) Upfront TMZ monotherapy (44, 45)	Persistent symptoms, high-risk features on MRI, rapid tumor progression, unsuitable for vorasidenib, PCV preferred due to OS benefit in 1p/19q-codeleted tumors

**Table 2:** Clinical management paradigm for grade 2 astrocytomas (IDH-mutant, non-1p/19q codeleted).

Risk Level	Risk Features	Treatment Options	Applicable Scenarios
Low Risk	Age<40 years,gross total resection(GTR),no uncontrolled symptoms, relatively slow growth rate (<8–10 mm/year, volume growth<10%or<8 cm³/year),may show minimal enhancement	Watchful waiting Vorasidenib (preferred) (2)	No uncontrolled symptoms(e.g., refractory seizures),non-enhancing or minimally enhancing residual disease(<1 cm),suitable for delayed RT/chemotherapy
High Risk	Uncontrolled symptoms(e.g., refractory seizures), contrast enhancement on MRI, significant residual tumor (>1cm),rapid growth (>10%or>8cm³/year), higher risk of malignant transformation	RT (54 Gy)+ sequential TMZ (preferred) (13) RT (54 Gy)+PCV (23) Upfront TMZ monotherapy (44, 45)	Persistent symptoms, high-risk features on MRI, rapid tumor progression, higher malignant transformation risk, unsuitable for vorasidenib, requires more frequent monitoring

## 8 Conclusion

Vorasidenib’s FDA approval as a first-in-class dual IDH1/2 inhibitor marks a significant advance in treating grade 2 IDH-mutant gliomas, underscored by the INDIGO trial’s substantial progression-free survival benefit. This targeted therapy holds the promise of delaying conventional treatments and improving quality of life. While current evidence for efficacy in grade 3 and 4 gliomas remains limited, ongoing research exploring combination strategies is crucial. Recent 2025 reviews emphasize the need for further investigation into vorasidenib’s role in higher-grade gliomas, particularly in pediatric populations, and its potential impact on seizure control, quality of life, and neurocognitive outcomes. Continued evaluation of long-term safety, optimal treatment timing, and broader application across glioma grades is needed, but vorasidenib signifies a transformative step in the multidisciplinary management of IDH-mutant gliomas.
